# Eales' disease - current concepts in diagnosis and management

**DOI:** 10.1186/1869-5760-3-11

**Published:** 2013-01-14

**Authors:** Jyotirmay Biswas, Reesha Karingattil Ravi, Angayarkanni Naryanasamy, Lily Therese Kulandai, Hajib Naraharirao Madhavan

**Affiliations:** 1 Department of Uveitis and Ocular Pathology, Vision Research Foundation, Sankara Nethralaya, No. 41, College Road, Nungambakkam, Chennai, 600006, India; 2 Little Flower Hospital and Research Centre, P.O. Box No. 23, Angamaly Kochi, Kerala, 683572, India; 3 Biochemistry Department, Vision Research Foundation, Sankara Nethralaya, No. 41, College Road, Nungambakkam, Chennai, 600006, India; 4 Department of Microbiology and Molecular Biology, Vision Research Foundation, Sankara Nethralaya, No. 41, College Road, Nungambakkam, Chennai, 600006, India

**Keywords:** Eales' disease, Human leukocyte antigen, Antioxidants, Retinal vasculitis, Photocoagulation, Polymerase chain reaction, Tuberculosis, Vitrectomy

## Abstract

Eales' disease, first described by the British ophthalmologist Henry Eales in 1880, is characterized by three overlapping stages of venous inflammation (vasculitis), occlusion, and retinal neovascularization. Diagnosis is mostly clinical and requires exclusion of other systemic or ocular conditions that could present with similar retinal features. In recent years, immunological, molecular biological, and biochemical studies have indicated the role of human leukocyte antigen, retinal autoimmunity, *Mycobacterium tuberculosis* genome, and free radical-mediated damage in the etiopathogenesis of this disease. However, its etiology appears to be multifactorial. The management depends on the stage of the disease and consists of medical treatment with oral corticosteroids in the active inflammatory stage and laser photocoagulation in the advanced retinal ischemia and neovascularization stages.

## Review

### Introduction

Eales' disease was first described by Henry Eales [1], a British ophthalmologist, in 1880 and 1882 who thought that it is a noninflammatory condition. The definition and etiology of Eales' disease are not adequately established [2]. In recent years, clinical and basic research have provided significant clues to the understanding of the clinical features and etiology of Eales' disease. Since the review article we published on Eales' disease in 2002, there has been tremendous advancement regarding the understanding of Eales' disease. This review aims to examine the current concepts of understanding of the etiopathogenesis and management of Eales' disease through clinical, biochemical, immunological, and molecular biological studies.

### Classification

Recently, Saxena and Kumar [3] have given a new staging system. The new system, according to the authors, is useful in classifying and assessing the severity of the disease (Table 1).

**Table 1:** New staging system for Eales' disease

**Stage**	**Description**
I	Periphlebitis of small (Ia) and large (Ib) caliber vessels with superficial retinal hemorrhages
IIa	Capillary nonperfusion
IIb	Revascularization elsewhere/of the disc
IIIa	Fibrovascular proliferation
IIIb	Vitreous hemorrhage
IVa	Traction/combined rhegmatogenous retinal detachment
IVb	Rubeosis iridis, neovascular glaucoma, complicated cataract, and optic atrophy

### Etiopathogenesis

**Figure 1:**
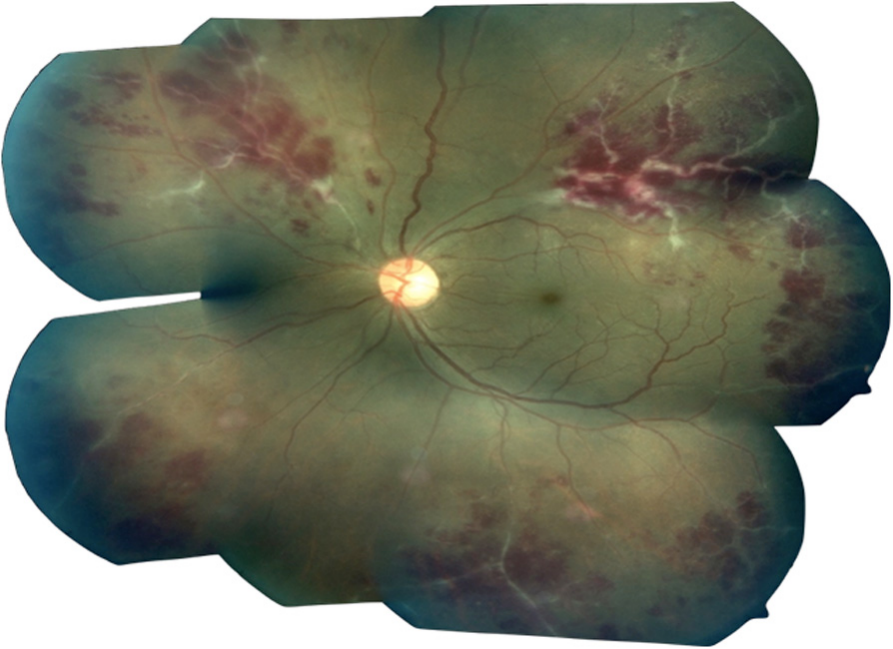
Fundus photograph of Eales' disease with active periphlebitis.

Of the several etiologies proposed, the most favored are tuberculosis and hypersensitivity to tuberculoprotein. In a retrospective study, 70% of epiretinal membrane (ERM) samples were positive for one or more *Mycobacterium* species tested by polymerase chain reaction (PCR). Further, we reported the presence of the *MPB64* gene of *Mycobacterium tuberculosis* in a significant number of ERMs of well-documented Eales' patients as compared with controls of well-documented non-Eales' patients [4]. PCR was found to be specific and sensitive enough to detect 2.5 pg of *M. tuberculosis* complex DNA. In another study, 11 out of 23 ERMs removed from the eyes of patients with Eales' disease showed *M. tuberculosis* genome by nested PCR technique [5]. However, culture of vitreous specimen did not show growth of *M. tuberculosis* in any of the vitreous aspirates*.* It appears that Eales' disease patients may not carry viable organisms, but may probably harbor nonviable organisms or DNA of *M. tuberculosis* in a significant number of cases. In a recent study, Singh et al. demonstrated MTB genome in more than 50% vitreous fluid samples with significant bacillary load. The authors concluded that many patients with the so-called Eales' disease could indeed have tubercular vasculitis, and therefore, it is important to investigate these patients for underlying tuberculosis [6]. The role of the *M. tuberculosis* genome in the pathogenesis of Eales' disease is yet to be ascertained. However, Eales' disease is not synonymous with the idiopathic periphlebitis (Figure 1). It accurses in certain geographic areas that have predisposition to certain age groups (15 to 40 years). Although it was described to be noninflammatory by Henry Eales, subsequently Elliot has described retinal vasculitis in Eales' disease [7]. Tubercular retinal perivasculitis is usually bilateral, with the presence of vitreous snowball opacities, neovascularization, retinal hemorrhage, neuroretinitis, and focal choroiditis [8].

Studies have demonstrated a statistically significant higher phenotype frequency of human leukocyte antigen (HLA) in Eales' disease patients than in healthy controls. It is hypothesized that individuals with a predisposition to HLA, predominantly HLAB5, DR1, and DR4, may develop retinal vasculitis as a consequence of cell-mediated immunologic tissue damage triggered by a sequestered mycobacterial antigen in an inactive form that clinically presents as Eales' disease (ED) [9].

### Pathology

Association of systemic inflammation with the inflammatory stage of ED has been established by the presence of elevated levels of C-reactive protein [10] or circulating interleukin (IL)-6 as a marker [11]. In ED patients, the number of circulating monocytes [12] was significantly more compared with that in controls, with a marked increase in the nonclassic CD16+ subset, which showed an activated phenotype in patients that correlated with levels of serum proinflammatory cytokines and clinical progression. A higher expression of cell surface toll-like receptor (TLR)-2, but not TLR-4, was found in monocytes of patients with ED [13]. Furthermore, TLR-2 was expressed at higher levels on CD16+ monocytes than on CD16− monocytes in ED patients, whereas no significant variation was found in TLR-4 expression on different monocyte subsets. Peptidoglycan-induced tumor necrosis factor alpha (TNF-α) expression correlated with TLR-2 expression in monocytes isolated from controls, but not in monocytes isolated from ED patients. This indicates that in the pathogenesis of ED, TLR activation and increased numbers of nonclassic CD16+ monocytes are crucial regulators, along with the secretion of proinflammatory cytokines that perpetuate the inflammatory process in the retina.

Oxidative stress has been shown by increasing levels of thiobarbituric acid-reactive substances (TBARS) in the vitreous, erythrocytes, platelets, and monocytes [14]. A decrease in vitamins E and C both in active and healed vasculitis, superoxide dismutase, glutathione, and glutathione peroxidase showed a weakened antioxidant defense. Free radical damage to DNA and of protein was shown by the accumulation of 8-hydroxy-2′-deoxyguanosine (in leukocytes) and nitrotyrosine (in monocytes) [15]. Nitrosative stress was shown by increased expression of inducible nitric oxide synthase in monocytes [16], in which levels of iron and copper were increased while those of zinc decreased [17]. A novel 88-kDa protein was found in serum and vitreous in an inflammatory condition and had an antioxidant function [18]. Increase in Fe^3+/^Fe^2+^ and iron-induced oxidative stress in Eales' disease was reported. With vitamin E and C supplementation, this ratio was significantly lowered [19].

A study was conducted to find out any relation existing between the levels of gelatinase and the cytokine TNF-α, known to upregulate matrix metalloproteinase (MMP) expression in various cells [20]. MMP-9 level and activity were found to be significantly higher in serum and vitreous samples of patients with Eales' disease than those of control subjects. Simultaneously, a positive correlation was found between intraocular TNF-α and MMP-9 concentration in patients with Eales' disease which provides a plausible explanation for inflammation-mediated angiogenesis during the development of this condition.

Pigment epithelium-derived growth factor (PEDF) is a glycoprotein and a potent inhibitor of ischemia-induced neovascularization. Decrease in PEDF is reported in the vitreous of proliferative diabetic retinopathy patients [21,22]. The ratio of the vitreous vascular endothelial growth factor (VEGF) and PEDF in Eales' disease was studied. The ratio of VEGF, an angiogenic factor, and PEDF, an antiangiogenic factor, in the vitreous of Eales' disease patients was compared with those of macular hole (MH) and proliferative diabetic retinopathy (PDR). The VEGF/PEDF ratio was found to be significantly increased in Eales' disease and PDR compared to MH. However, the ratio was 3.5-fold higher in PDR than in ED. A case report on the IHC of an enucleated ball of an Eales' disease patient revealed a strong positive staining for PEDF, in comparison to an age- and sex-matched donor eyeball [23].

Retinal S-antigen and interphotoreceptor retinoid-binding protein-3 are found to play a significant role in the etiopathogenesis of Eales' disease. Three-dimensional (3D) protein structures are functionally very important and play a significant role in the progression of the disease; hence, these 3D structures are a better target for further drug designing and relative studies [24].

### Management

Patients with inactive retinal vasculitis can be observed periodically at 6-month to 1-year intervals. Patients with fresh vitreous hemorrhage also are asked for observation at intervals of 4 to 6 weeks if the underlying retina is found to be attached by indirect ophthalmoscopy or by ultrasound. Such vitreous hemorrhage often clears by 6 to 8 weeks.

### Medical therapy

Corticosteroids remain the mainstay of the therapy in the active perivasculitis stage of Eales' disease. As many investigators believe that hypersensitivity to tuberculoproteins plays a role in the etiology of Eales' disease (Mantoux positive), antitubercular treatment is given for a period of 9 months. Retinal periphlebitis with strongly positive PPD test requires the use of systemic steroids and appropriate antituberculous therapy even in the absence of active systemic disease to avoid reactivation of the systemic illness. Abu El-Asrar and Al-Kharashi found in a study of 30 patients that aggressive treatment of Eales' disease with systemic steroids and antituberculous therapy, full panretinal photocoagulation and early vitrectomy, when necessary, may result in the improvement of the anatomic and visual outcome [25]. Macular involvement is uncommon in Eales' disease but can occur with central or extensive disease and in the late stages of proliferation. Periocular depot corticosteroid/triamcinolone acetonide injections are given in cases with macular edema [26]; these injections have been found to be beneficial, avoiding permanent damage to the macula. Because the corticosteroids usually work very well in the acute inflammatory stage of Eales' disease, steroid-sparing immunosuppressive agents like cyclosporine and azathioprine are reserved for patients for whom corticosteroids are not effective or must be discontinued and/or are contraindicated because of their side effects.

Oxidative damage to cellular membranes plays an important role in the pathobiology of tissue injury. A study was undertaken to study the effect of antioxidant supplementation over membrane fluidity in platelets in idiopathic retinal periphlebitis (Eales' disease) [27,28]. Assay of TBARS levels was done following a standard protocol, and membrane fluidity in platelets was estimated using a fluorescent probe, in 15 cases and 12 healthy controls [11]. Pre- and post-antioxidant supplementation and platelet TBARS and membrane fluidity levels were assessed in all the cases. A significant increase was observed in TBARS levels in the cases when compared with the controls.

In addition to oxidative stress, hyperhomocysteinemia is also reported in Eales' disease [29]. If hyperhomocysteinemia is found in the patients, it could also cause oxidant assault. In such cases, a daily dose of folate (1 to 2 mg) and vitamin B_12_ (500 μg) orally will be beneficial and be continued until homocysteine levels are normalized. Another study was by Saxena et al. to know the effect of oral antioxidant supplementation in Eales' disease. Antioxidant supplementation (vitamins E, C, and A; beta carotene; Cu; Zn; and Se) was given for 3 months. Statistically significant differences were observed in TBARS, SOD, and GSH but not catalase [28]. Antioxidants that harmoniously neutralize harmful oxidants were evaluated as serum total antioxidant capacity (TAC) in Eales' disease which was significantly lowered as compared to healthy adults. The lowering was more in active vasculitis cases of Eales' disease than the quiescent stage. With vitamin E and C supplementation, TAC levels improved significantly [30].

Raised levels of IL-1β and TNF-α were observed in the inflammatory stage and persisted in the proliferative stage of the disease. The IL-1 system and TNF-α represent a novel target for immunotherapy for controlling inflammatory activity and/or the associated long-term sequelae related to angiogenesis in Eales' disease [11].

### Intravitreal injections

Indeed, the most common presentation of Eales' disease is a sudden painless loss of vision because of vitreous hemorrhage [31]. The usual management is initially conservative, and vitrectomy is reserved for eyes with nonresolving vitreous hemorrhage at the end of 3 months [32]. Timely regression of new retinal vessels is desired to avoid vitreous hemorrhage and tractional retinal detachment requiring vitreoretinal surgery [33-36]. It has been suggested that the massive VEGF expression observed in the retinal neovascular membranes may be due to retinal ischemia and chronic low-grade inflammation. Good results and adequate control of inflammation with the use of intravitreal triamcinolone in patients with Eales' disease have been reported previously. However, common complications such as elevated intraocular pressure and cataract progression may limit the use of intravitreal steroids. In a recent study from India, where the disease is relatively common, the beneficial effect of intravitreal bevacizumab in a patient with presumed Eales' disease has been reported [37]. Fundus examination in a patient with vitreous hemorrhage secondary to Eales' disease often shows a combination of both fresh and old vitreous hemorrhage at any time the patient is examined, thereby suggesting that there may be bleeding on more than one occasion in these patients. Also recently, bevacizumab has been shown to be effective in regressing neovascularization and mild hemorrhage secondary to Eales disease, if given every 4 weeks, thereby hastening the process of resolution of dense vitreous hemorrhage [35] or reduce the need for vitrectomy.

### Photocoagulation

Photocoagulation is the mainstay of treatment in the proliferative stage of Eales' disease. As vitreous hemorrhage could occur at any stage of Eales' disease, we are unclear whether laser photocoagulation could be beneficial in the inflammatory stage of the disease. It is also unclear whether panretinal photocoagulation involving all four quadrants is necessary, similar to diabetic retinopathy, or a segmental scatter photocoagulation of the involved quadrants of the retina, similar to branch retinal vein occlusion, will suffice. Fluorescein angiography helps in monitoring the response to treatment. A study to evaluate the usefulness of prophylactic scatter photocoagulation in asymptomatic eyes of patients presenting with vitreous hemorrhage due to Eales disease revealed that prophylactic photocoagulation is an effective method of controlling the secondary complications in asymptomatic eyes of patients with Eales' disease especially if managed at an early stage [38].

The balance between VEGF and PEDF has been implicated in the mechanism of angiogenic ocular diseases. One of the observations in the study by Angayarkanni et al. on the effect of steroid therapy on the VEGF/PEDF ratio in Eales' disease cases was that the interval between last steroid intake and time of vitrectomy showed a lowered ratio in those receiving steroids between 1 and 6 months preceding surgery as compared to those receiving steroids in the month immediately preceding surgery. Moreover, the VEGF levels in the sectoral laser treatment group were significantly higher than those in the no-laser group, and the ratio of VEGF/PEDF was also higher in spite of the increased PEDF levels. The sectoral laser is done when the disease is relatively still active, while in the panretinal photocoagulation (PRP) group, the disease in the involuntary stage. The PRP group showed a ratio similar to that of the no-laser group [23].

### Surgery

Recurrent vitreous hemorrhage is the major cause for poor visual outcome in Eales' disease. Early posterior vitreous detachment that usually occurs in these eyes helps the surgeon in getting into the right plane for a safe vitreous surgery. Application of endolaser is mandatory at the conclusion of vitreous surgery. Additional procedures, such as belt buckling and lensectomy, are occasionally required. Uncomplicated pars plana vitrectomy has shown improvement of visual acuity in majority of patients. The major postoperative complications are early development of cataract, secondary glaucoma, and rhegmatogenous retinal detachment [39]. Usefulness of pars plana vitrectomy in managing asymptomatic eyes of Eales' disease patients was studied [40], and the study concluded that early vitrectomy in established cases of Eales' disease provides satisfactory results and helps in preventing complications, which are difficult to treat.

## Conclusions

None of the recent studies could differentiate Eales' disease from tuberculous vasculitis, and also several studies, especially from India where the disease is prevalent, reported the strong association between this type of occlusive vasculitis and tuberculosis. Therefore, it is better to name this disease as presumed tuberculous retinal periphlebitis and should be treated with full antituberculous therapy plus a short course of systemic corticosteroids. Scatter laser photocoagulation is indicated in patients with features of retinal ischemia with new vessels. Early pars plana vitrectomy is indicated in patients with vitreous hemorrhage. Those cases where the work-up is negative can be termed as idiopathic occlusive vasculitis. Thus, we conclude that the name of the disease Eales' disease will no longer exist.

## Competing interests

The authors declare that they have no competing interests.

## Authors’ contributions

JB, RKR, AN, and LTK wrote the manuscript. JB, AN, LTK, and HNM reviewed the manuscript. RKR collected the references. All authors read and approved the final manuscript.
